# Supplementary material for: Adherence to antiretroviral therapy and associated factors among Human immunodeficiency virus positive patients accessing treatment at Nekemte referral hospital, west Ethiopia, 2019
Source: PLoS One. 2020 May 11;15(5):e0232703. doi: 10.1371/journal.pone.0232703 (PMC7213685; doi:10.1371/journal.pone.0232703)
Supplement: S1 Questionnaire — (DOCX) [file pone.0232703.s001.docx]

Wollega university

Institute of health sciences

School of nursing and midwifery

Department of nursing

**Questionnaires (English version)**

**Consent form**

This questionnaire is prepared to collect data on **“Adherence to antiretroviral therapy and associated factors among Human immunodeficiency virus-positive patients accessing treatment at Nekemte referral hospital, West Ethiopia, 2019”.** You have been included randomly to this study and the information you will provide, will neither be disclosed to the third party, nor be used for another purpose and will be rather kept confidential. The information collected will not be linked to you or your family members and you don’t need to tell your/family member/ name. You have full right to participate, reject or not to answer all or part of the questions. However, your participation in giving the right information is quite important for our study.

Do you agree to participate?

Yes Continue the interview

No Thank him or her

Name of interviewer

Signature

Crosschecked by the supervisor A/yes B/No Signature

**Part I: Sociodemographic characteristics of the study participants**

1. What is your gender?
2. Male B. Female
3. What is your age in years? _______________
4. What is your current marital status?
5. Married C. Divorced
6. Single D. Widowed
7. What is your ethnicity?
8. Oromo D. Gurage
9. Amhara E. Others
10. Tigre
11. What is your current educational status?
12. No formal education
13. Primary school (1-8)
14. Secondary school (9-12)
15. College and above
16. What is your current residence or where do you live now?
17. Urban B. Rural
18. What is your occupation?
19. Government employee
20. Private employee
21. Farmer
22. Merchant
23. Others
24. What is your average monthly income in Ethiopian birr? ________
25. Have you a living companion?
26. Yes B. No

**Part II: Clinical and social/behavioral related characteristics of the study participants**

1. How many pills you take per day?
2. 2 tablets C. 4 tablets
3. 3 tablets D. >4 tablets
4. What is the patients CD4 level (To be filled from patient cards) ________?
5. What is the patient’s HIV stage (To be filled from patient cards)?
6. Stage I
7. Stage II
8. Stage III
9. Stage IV
10. Do you use substance? Tick “yes” if the patient use at least one of these substances: Alcohol, illegal drugs, chat, Heroin, cocaine, marijuana, cigarettes and other tobacco products.
11. Yes B. No
12. How many years since you acquired the disease?
13. <1 year
14. 1-5 years
15. 6-10 years
16. >10 years
17. How many years since you started the treatment?
18. <1 year
19. 1-5 years
20. 6-10 years
21. >10 years
22. Have you a comorbidity of other chronic illness? (Tick yes if the patient has at least one of any type of chronic illness)
23. Yes B. No
24. Have you disclosed your HIV status to your families and friends?
25. Yes B. No
26. What is the average waiting time to get treatment?
27. <30 minutes
28. >/=30 minutes

**Part III: Assessment of Side effect of ARV drugs**

1. Have you experienced any of the side effects of ARV medication listed below?

Loss of appetite Rash or hypersensitivity

Lipodystrophy Fatigue

Nausea and vomiting Trouble sleeping

Diarrhea Numbness in hands and legs

Mood changes Sign and symptoms of anemia

If the patient experienced at least one of the side effects listed above, tick yes as the response.

1. Yes B. No

**Part IV: Assessment of Family/social support**

1. How many people are so close to you that you can count on them if you have great personal problems?
2. None
3. 1-2
4. 3-5
5. >5
6. How much interest and concern do people show in what you do?
7. None
8. Little
9. Uncertain
10. Some
11. A lot
12. How easy is to get practical help from neighbors if you should need it?
13. Very difficult
14. Difficult
15. Possible
16. Easy
17. Very easy

Note: Consider “poor support” if the range is 3–8, “moderate support” if the range is 9–11 and “strong support” if the range is 12–14.

**Part V: Perceived social stigma assessment scale**

1. Some people avoid touching me once they know I have HIV
2. Strongly disagree
3. Disagree
4. Agree
5. Strongly agree
6. Telling someone I have HIV is risky
7. Strongly disagree
8. Disagree
9. Agree
10. Strongly agree
11. I have lost friends by telling them I have HIV
12. Strongly disagree
13. Disagree
14. Agree
15. Strongly agree
16. I work hard to keep my HIV secret
17. Strongly disagree
18. Disagree
19. Agree
20. Strongly agree
21. I am very careful who I tell that I have HIV
22. Strongly disagree
23. Disagree
24. Agree
25. Strongly agree
26. People I care about stopped calling after learning I have HIV
27. Strongly disagree
28. Disagree
29. Agree
30. Strongly agree
31. Most people are uncomfortable around someone with HIV
32. Strongly disagree
33. Disagree
34. Agree
35. Strongly agree
36. I feel guilty because I have HIV
37. Strongly disagree
38. Disagree
39. Agree
40. Strongly agree
41. I feel I am not as good a person as others because I have HIV
42. Strongly disagree
43. Disagree
44. Agree
45. Strongly agree
46. People’s attitudes about HIV make me feel worse about myself
47. Strongly disagree
48. Disagree
49. Agree
50. Strongly agree
51. People with HIV are treated like outcasts
52. Strongly disagree
53. Disagree
54. Agree
55. Strongly agree
56. Most people believe that a person who has HIV is dirty
57. Strongly disagree
58. Disagree
59. Agree
60. Strongly agree

Note: A sum score is calculated with a minimum score of 12 and maximum score of 48, with a higher scores reflect a higher level of perceived HIV related stigma. The overall HIV stigma scale ranged from 17 to 38, while the mean score was 28.34 with +/- 9.25 standard deviation (95% CI: 25.21, 30.42). Then, the mean score of 28.34 was used to categorize the patients as stigmatized or not stigmatized. Accordingly, 180 (59.0 %) of the study participants don’t experienced social stigma and the remaining 125 (41.0 %) had experienced social stigma.

**Part VI: Patients Knowledge about HIV and its treatment**

1. Is ART reduce HIV related morbidity?
2. Yes B. No
3. Is ART reduces HIV related mortality?
4. Yes B. No
5. Is HIV is controlled by ART?
6. Yes B. No
7. Does patient trust the doctor?
8. Yes B. No
9. Do you know how to deal with side effects?
10. Yes B. No
11. Do you stops taking ART on side effects without health professionals’ consultation?
12. Yes B. No
13. Do you know the effectiveness of ART?
14. Yes B. No
15. Do you think not abiding to ART leads to drug resistance?
16. Yes B. No

**Note:** The knowledge score was categorized into two levels indicated by poor knowledge/not knowledgeable (0-4) and good knowledge/knowledgeable (5-8).

**Part VII: Assessment of level of adherence to antiretroviral medication**

**Adherence worksheet**

Patient code______________

1. Number of tablets dispensed at last visit_______
2. Number of tablets returned at this visit (count the tablets the patient has brought) _____
3. Number of days since last visit_________
4. Number of tablets client takes per day_____________
5. Number of tablets client actually used_________
6. Number of tablets patient should have taken___________

Percentage of adherence = Number of tablets client actually taken divided by umber of tablets patient should have taken x 100

**Note:** Patients who reported intake of ≥ 95% of the prescribed medication were considered adherent; and those with a reported intake of < 95% were classified as non-adherent.

Thank you for your participation
