# Supplementary material for: Adherence to antiretroviral therapy and associated factors among Human immunodeficiency virus positive patients accessing treatment at Nekemte referral hospital, west Ethiopia, 2019
Source: PLoS One. 2020 May 11;15(5):e0232703. doi: 10.1371/journal.pone.0232703 (PMC7213685; doi:10.1371/journal.pone.0232703)
Supplement: Attachment — Submitted filename: Response to reviewers.docx [file pone.0232703.s004.docx]

Date: 22/12/2019

**To PLOS ONE**

Dear Academic Reviewers,

**Subject:** Submission of revised Manuscript **“Adherence to antiretroviral therapy and associated factors among Human immunodeficiency virus-positive patients accessing treatment at Nekemte referral hospital, West Ethiopia, 2019”.** Thank you very much for reviewing our manuscript. We also greatly appreciate the reviewers for their constructive comments and suggestions. We have carried out the revisions that the reviewers requested and revised the manuscript accordingly. We hope the revised version is now suitable for publication and look forward to hearing from you in due course. Point-by-point responses are attached hereafter this page.

Sincerely,

Muktar Abadiga

Corresponding author

Wollega University, Ethiopia

**Response to reviewers**

**Reviewer #1**

Background

1. Inclusion of a brief description of key elements of ART services offered at the hospital can make reader understand the context better.

**Response:** This is a very important comment and we included this information in the revised version of our manuscript.

Sample size determination and sampling techniques

1. Brief description of the sampling method would help reader to understand and determine if there were on potential sources of bias that can affect results and interpretation. What was the sampling framework – ART records at the facility or patients were randomly selected as they came for their clinic visit during the study month?

**Response:** There were about 2251 HIV/AIDS patients on treatment follow up at Nekemte referral hospital at time of study. The patients were randomly selected as they came for their hospital visit during the study period. A simple random sampling method was used to select 311 study participants to be involved in the study. These all informations are included in this revised version of our manuscript.

2. 311 patients should be estimated sample size for the study

**Response:** Yes, 311 patients were the estimated sample size for the study. Initially, a total of 311 study participants were sampled to be participated in the study. Out of these 311 study participants, six participants were eligible for the study but not willing/refused to take part in the study. Therefore, they were excluded from participating in the study, and only 305 study participants were participated in the study. The detail explanations are included in this revised version of this manuscript.

3. It is not clear if 311 patients represent total number of patients at the facility, in the study design and setting section, “All ART patients on treatment follow up at Nekemte referral hospital was the source population and the sampled ART patients who had treatment follow up during the study period was the study population”, It would be easy to follow the sampling methodology if total number of patients on ART is mentioned and how random sampling was applied.

**Response:** The total number of HIV/AIDS patients having treatment follow up at Nekemte referral hospital was 2251 which is a source population. 311 patients were the estimated sample size for the study after sample size calculation. From this 311 participants, 305 study participants were participated in the study (6 participants) refused to participate. These 305 participants were selected by using simple random sampling techniques as they came for their hospital visit during the study period. These informations are also included in this revised version of this manuscript.

Ethical consideration

“Filled out questionnaires were carefully handled and all access to results was kept strictly within the author” brief description how records were handled and stored can help the leader to understand instead of just “carefully handle and kept strictly by author”

**Response:** We mean that immediately after the patients’ interview, the principal investigator collects the questionnaire from data collectors and stored where no one can have access to the questionnaires. Therefore, the questionnaire was not exposed to third parties and the patients’ information was kept confidential.	This is modified in the revised version of this manuscript.

Results

311 sampled - state out of how many patients in total.

**Response:** There were about 2251 HIV/AIDS patients on treatment follow up at Nekemte referral hospital at time of study. Out of this 2251 patients, 311 were sampled to be participated in the study. We mistakenly didn’t indicate this total number of patients on treatment follow up in the previous version of our manuscript. But, we indicated in this revised version of our manuscript.

How were social support categories derived?

**Response:** Oslo 3-item social support scale were used to measure social support. These items are:

1. How many people are so close to you that you can count on them if you have great personal problems?
1. None
2. 1-2
3. 3-5
4. >5
2. How much interest and concern do people show in what you do?
1. None
2. Little
3. Uncertain
4. Some
5. A lot
3. How easy is to get practical help from neighbors if you should need it?
1. Very difficult
2. Difficult
3. Possible
4. Easy
5. Very easy

The OSS-3 scores ranged from 3-14. Oslo 3-item social support scale has three categories: “Poor support” if the range is 3–8, “Moderate support” if the range is 9–11 and “Strong support” if the range is 12–14.

What substances were used?

**Response:** According to our study, substance use includes the use of substances such as alcohol, illegal drugs, chat, Heroin, cocaine, marijuana, cigarettes and other tobacco products. In this study, the patient is considered as using substance if he/she is currently using any one of the above mentioned substances. The detail explanations are included in this revised manuscript.

WHO clinical staging and CD 4 count levels- explain when these were determined for the patients as they are likely to be influenced by ART. It is not clear if this was the patient’s status at the time of the study or at the time of the ART initiation. Or how recent?
**Response:** WHO clinical staging of HIV/AIDS is performed each month the patient visit the clinic according to the hospital in which this study was conducted. Therefore, the WHO clinical stages of HIV/AIDS mentioned in this study was taken from the patients card which was determined by health professional working at ART clinic. So, it was the WHO HIV/AIDS stage at the time of study. However, CD4 count is performed every 3 months in this hospital and the most recent CD4 count was taken for this study.

General comment

The author should ensure the grammatical errors are corrected throughout the manuscript.

**Response:** We agree that the previous version of our manuscript has some grammatical and editorial problems. We used free online grammar correction to solve this problem in this revised version of our manuscript. Therefore, the grammar of our revised manuscript is now improved.

Thank you very much!

**Reviewer #2**

General comments

The author addresses a very important public health problem. As already stated by the author, ART adherence is a determining factor as far as the fight towards rolling-back the HIV/AIDS pandemic is concern. In this write-up, the author tries to determine the adherence status of patients on HAART and goes further to identify factors influencing adherence. The write-up is complete with all the required sections but still has some faults. The English in the whole write-up needs to be carefully corrected by a native English speaker or an expert. Some vital information is missing in the background and methodology section. In addition, more clarifications are needed in some sections.

**Response:** Thank you very much for your constructive and crucial comments. We agree that the previous version of our manuscript has some grammatical and editorial problems. We used free online grammar correction to solve this problem in this revised version of our manuscript. Therefore, the grammar of our revised manuscript is now improved. The information missed in the previous submission of our manuscript is also corrected in this revised manuscript.

Title: Please do replace the word “attending” with “accessing”

**Response:** We accepted this comment and replaced our former title with **“**Adherence to antiretroviral therapy and associated factors among Human immunodeficiency virus positive patients accessing treatment at Nekemte referral hospital, west Ethiopia, 2019”.

Abstract

1. Please have your abstract read by a native English speaker and correct the errors

**Response:** We agree that the previous version of our manuscript has some grammatical and editorial problems. As we mentioned above, we used free online grammar correction to solve this problem in this revised version of our manuscript. Therefore, the grammar of our revised manuscript is now improved

2. Please do precise in what direction each of the cited factors affects adherence to treatment.

**Response:** We mistakenly didn’t show the direction of association of different independent factors with adherence in the previous version of our manuscript. So, we showed the direction of association of independent variables with dependent variable in this revised version of our manuscript. It is indicated as follows: Having knowledge about HIV and its treatment (Adjusted odd ratio=8.13, 95% Confidence interval: 3.06, 21.61), strong family/social support (Adjusted odd ratio= 7.36, 95% Confidence interval: 2.07, 26.10), absence of adverse drug reactions (Adjusted odd ratio = 5.62, 95% Confidence interval: 2.11, 14.93), no comorbidity of other chronic diseases (Adjusted odd ratio = 5.46, 95% Confidence interval: 1.86, 16.02) and disclosing HIV status to the family (Adjusted odd ratio=5.27, 95% Confidence interval: 2.20, 12.62) were significantly associated with increased likelihood of adherence to antiretroviral medication among HIV/AIDS patients. The detail description of these associations are included in this revised version of our manuscript.

3. What does the author mean by WHO standard? Please change “WHO standard” to “WHO recommendation”.

**Response:** We mean WHO recommendation. So, we accepted this comment and changed “WHO standard” to “WHO recommendation” in this revised version of our manuscript.

Background
The background encloses a lot of grammatical errors that need to be addressed carefully. Practically every sentence needs an English correction that I can’t go citing one after the other. This section is poorly organized with a lot of repetitions. I think in this section, the reader will want to know what ART is, and the importance of this treatment, what ART adherence is, current global findings on the status of adherence globally, in Africa and more specifically Ethiopia, what are the consequences of poor ART adherence and the impeding impact on the triple 90 ambitions set by the WHO, what are some of the factors registered in studies to influence ART adherence? And then you end with a paragraph on why the present study was necessary. Please do reorganize this section accordingly.

**Response:** These are also a very important comments and we addressed them in this revised version of our manuscript.

4. In the last paragraph of your background. “Since most of the Ethiopian setting is resource limited, there are no routine assessments for ART adherence among PLHIV in Ethiopia”. I don’t see any cause effect relationship in the two parts of this statement. In addition, I think in all HIV treatment centers routine follow-up ART adherence is done at the level of the center.

**Response:** We mean that Ethiopia is one of the developing countries which has inadequate resources. Therefore, routine assessment of ART adherence among PLHIV is not conducted at the hospital level due to resource constraints. However; we decided to remove this statement from the paragraph and we removed it in this revised version of our manuscript.

5. The fact that studies are few does not justify your work. Studies might be few but present enough evidence in that domain. I think that with changes in HIV related strategies, changes in the sociodemographic and economic status, with the routine interventions to combat HIV related stigma and discrimination, with the recent adoption of the test-and-treat strategy……etc, evaluative studies to mark progress on the status of ART adherence and possible associated factors (which might have changed over time) are necessary.

**Response:** As we said in the previous submission of our manuscript, only a few studies have been done on the adherence status of ART and its determinant factors in Ethiopia and no study was done particularly in this study area (Nekemte referral hospital). Many of the studies conducted on this issues in Ethiopia were conducted in the Southern, Northern and Eastern parts of Ethiopia. None of the studies were conducted in Western parts of the country specifically in Nekemte referral hospital. The following reasons were also considered before conducting this study at Nekemte referral hospital. These are**:**

i) Availability of enough population to enroll in the study. Nekemte referral hospital is the largest hospital in western part of Ethiopia with 2251 HIV/AIDS patients on treatment follow up. This number of HIV/AIDS patients in this hospital is much higher than any other hospitals found in western part of Ethiopia. So, selection of this hospital enhances availability of enough and eligible participants for the study.
ii) The second reason for selection of this hospital is proximity and accessibility of this hospital for data collectors, supervisors as well as for principal investigator to monitor the overall research project.
iii) The third reason for selection of this hospital was there were no competing demands or no other studies that would interfere with this study. In other words, there was no participation of study participants in other studies with similar inclusion criteria which might interfere with this study.
iv) The fourth reason was absence of similar studies conducted in the study area. Even though the number of HIV/AIDS patients on treatment follow up in this hospital is high, nothing is known about adherence of HIV/AIDS patients to ART. Therefore, the reasons mentioned above were taken into account to conduct this study in this hospital.
6. Please also do reformulate with more precision the objective of this study presented at the end of this section. Please end with a full stop.

**Response:** We accepted this comment and reformulated as follows: “This study was aimed to assess adherence status and associated factors among HIV infected patient having treatment follow up at Nekemte referral hospital.”

Methods
This section lacks some very important precisions and the English errors persist.

**Response:** The methods part is now written with precision and the grammar of our revised manuscript is now improved.

7. Nekemte referral hospital. Nothing is said on the treatment center in this hospital. What rational have you for choosing this hospital for your study? How many patients does this center follow up monthly? How many workers found in the center and what are their qualifications? Are all these workers trained?

**Response:** Nekemte referral hospital is found in Nekemte town and is one of the largest towns found in western part of Ethiopia at about 325 kilometers from a capital city of Addis Ababa. There were about 2251 HIV/AIDS patients on treatment follow up at Nekemte referral hospital at time of study. There were no similar studies conducted in the study area. Even though the number of HIV/AIDS patients on treatment follow up in this hospital is high, nothing is known about adherence of HIV/AIDS patients to ART. Regarding the number of workers in this hospital, there were 21 medical doctors, 57 nurses, 42 midwives and 13 pharmacist working in this hospital during this study. All the above informations were included in this revised version of our manuscript.

8. Refusal to participate is an ethical issue and not an exclusion criterion. In addition, were newly diagnosed patients included? What about those who arrived critically ill?

**Response:** Refusal to participate in the study was not considered as an exclusion criterion in our study. We considered it as non-response rate, that is why our response rate is only 98.07 %. But, the way we wrote this in our previous submission was not appropriate and we removed it in this revised version of our manuscript. We haven’t come across with newly diagnosed patients, and no patient was arrived critically ill during the data collection.

9. How were participants included in this study? I guess that data collectors were stationed at the treatment center. How was your simple random sampling implemented?

**Response:** The data collection was undergone at ART clinic of Nekemte referral hospital and the study participants were randomly selected using lottery methods (simple random sampling using lottery methods). The randomly selected patients were interviewed at isolated office to maintain privacy. The detail explanations were included in this revised version of our manuscript.

10. Were the data collectors’ workers at the HIV unit? Or did they come to the unit just to do data collection? How were potential eligible participants identified? What strategies were used to reduce stigmatization associated to the new team if they were new?

**Response:** Data collectors were recruited from nurses working in Gimbi general hospital which is found at about 145 km from Nekemte referral hospital. They came to this Nekemte referral hospital just to do data collection. The data collectors were health professionals and they know professional ethics. Therefore, stigmatization might not be the critical issue. In addition, the nurses (data collectors) were recruited from another hospital and they don’t know the patient personally to stigmatize them.

11. The design used in this study is disturbing except clarifications will come. Were blood samples of the participants collected for work ups (case of CD4 for instance) or was there any documentary or register review? If all was done by interview, how did you verify the information?

**Response:** In this study, data was collected using a structured questionnaire and face-to-face interview was used for data collection. In addition to the interview, other patient informations were obtained from patient cards. For example: Information such as CD4 level and WHO staging of HIV/AIDS were obtained from the patients’ cards. WHO clinical staging of HIV/AIDS is performed each month the patient visits the clinic and was taken from the patients card which was determined by health professional working at ART clinic. CD4 count is performed every 3 months in this hospital and the most recent CD4 count was taken for this study from the patients’ cards. These informations are also included in the methodology section of this revised manuscript.

12. The procedure of implementation and the data collection procedure is scanty. Were any administrative procedures undertaken? How did data collectors approach eligible participants? Were interviews done behind closed doors? Etc

**Response:** The patients who fulfilled the inclusion criteria were randomly selected (simple random sampling using lottery methods) as they came for their hospital visit during the study period. Then, the randomly selected patients were brought to private room and the interview was conducted behind the closed doors after the consent was received. The patients were interviewed one by one (one patient at a time) in the isolated room to maintain privacy. These informations are also included in the methodology section of this revised manuscript.

13. The data processing and analysis section is very scanty and cannot be reproduced. The result section presents two p-values….0.25 and 0.05 for which this is not mentioned in this section. How were variables included into the multivariable regression analysis?

**Response:** Bivariate analysis using logistic regression was done to find association between each independent variable with adherence to antiretroviral medication. All independent variables were entered into multivariable logistic regression for further analysis. Finally, multivariable logistic regression with backward elimination was used to find out the independent variables which best predict patient’s adherence to antiretroviral medication. Therefore, P values of 0.25 was used to determine significance at bivariate level and 0.05 was used to determine significance at multivariate level. However; all association and statistical significance were measured using odds ratio at 95% confidence interval and p-value of less than 0.05. These all are illustrated in this revised version of our manuscript.

14. The data control section logically should be presented before analysis. In addition, what was the content of the one-day training delivered to data collectors and supervisors? Were supervisors and data collectors given the same training?

**Response:** These are also a very important comments and we incorporated the correction in the revised version of this manuscript. The content of the training for data collectors and supervisor are different and given separately for them. “One-day training on questionnaire items, content of the questionnaire, how the data to be collected and the patient approach was given for data collectors. The supervisor was also trained on how to monitor data collection, checking the completeness of the questionnaire immediately after collection and how to handle and store the filled questionnaire.”

15. Clearly state in the ethical consideration section. What type of consent was obtained from participants?

**Response:** As we indicated in the previous version of our submission, the written informed consent was obtained. This was indicated under the heading ethical consideration as follows: “All participants of the study were provided written consent, clearly stating the objectives of the study and their right to refuse, and written informed consent was obtained from the participants.”

16. The data collection tool should be made available for publication to ease reproducibility.

**Response:** The questionnaire used in this study includes socidemographic characteristics, psychosocial and medication related characteristics or clinical and behavioral measurement questionnaire, measurement of adherence scale, Oslo 3-item social support scale and Disease/treatment knowledge. We used Pill counting method, which is an objective method for determining adherence in this study. After the identification of the hospital in which this study was conducted, we communicated with the health professionals to inform the HIV/AIDS patients on treatment follow up to bring the medication bottle or strips dispensed during the next subsequent visit. So, patients were asked to bring residual medication at next subsequent appointments. This was done before the actual period of data collection, and the medication bottle or strips dispensed during the previous visit were brought by the patient. The data collectors were also assured that patients didn’t buy/sell or borrow/give medications from/to others to get by the pill count. The number of pills taken is calculated by subtracting the count of the number of pills remaining from the total number of pills dispensed. Then, the adherence rate was calculated by dividing the number of pills the patient actually taken by number of tablets patient should have taken x100. The following are adherence worksheet we used.

**Adherence worksheet**

Patient code______________

1. Number of tablets dispensed at last visit_______
2. Number of tablets returned at this visit (count the tablets the patient has brought) _____
3. Number of days since last visit_________
4. Number of tablets client takes per day_____________
5. Number of tablets client actually used_________
6. Number of tablets patient should have taken___________

Percentage of adherence = Number of tablets client actually taken divided by umber of tablets patient should have taken x 100

Finally, based on WHO Guideline, Patients who reported an intake of ≥ 95% of the prescribed medication were considered adherent; and those with a reported intake of < 95% were classified as non-adherent.

Disease/treatment knowledge was measured using eight questions taken from previous similar studies: ART reduces HIV related morbidity? ART reduces HIV related mortality? HIV is controlled by ART? does patient trusts the doctor? do you knows how to deal with side effects? do you stops taking ART on side effects without health professionals’ consultation, do you know the effectiveness of ART? and not abiding to ART leads to drug resistance? This ART knowledge score was calculated as a continuous variable by summing the participant’s number of correct responses to 8 statements. One point was awarded for each correct response (Yes or No for correct statement), and zero for each wrong response, with a maximum obtainable correct score of 8 for each respondent. The knowledge score was categorized into two levels indicated by poor knowledge/not knowledgeable (0-4) and good knowledge/knowledgeable (5-8).

Oslo 3-item social support scale were used to measure social support. The OSS-3 scores ranged from 3-14. Oslo 3-item social support scale has three categories: “Poor support” if the range is 3–8, “Moderate support” if the range is 9–11 and “Strong support” if the range is 12–14.

All the questionnaires used in this study are available from the correspondence author on request. We also sent you this tools as a separate file in this revised version of our manuscript.

Results
Please do correct the English in this section. In general, the confidence intervals of the odd ratios are very large indicative of small cell number as far as the considered variables are concerned.

**Response:** We agree that the previous version of our manuscript has some grammatical and editorial problems. We used free online grammar correction to solve this problem in this revised version of our manuscript. Therefore, the grammar of our revised manuscript is now improved.

17. Looking at your age distribution, it seems skewed and therefore the best measure of central tendency is the median.

**Response**: We agree that the age distribution is slightly skewed to the right (positively skewed). In this age distribution, the mean is greater than the median (mean = 32.63 and median = 31.00). As a reviewer mentioned, the best measure of central tendency when the datas are skewed is median. In this study, the minimum age of the study participants was 18 years and maximum age was 56 years. Therefore, the median age of the study participant was 31.00 years with a range of 38 years (56 years-18 years). Therefore, we modified this in the revised version of our manuscript.

18. How did you clinically stage your participants only by interview?

**Response:** The data collectors didn’t clinically stage the patient by interview for the purpose of this study. As we discussed in the comment number 11, the WHO clinical staging of HIV/AIDS is performed each month the patient visits the clinic and was taken from the patients card which was determined by health professional working at ART clinic. Therefore, it was not determined only by interview, and therefore it was taken from the patients’ cards.

19. How did you measure knowledge on HIV and treatment? Please clearly state how this was done on the methods section. Who had good knowledge and who poor knowledge?

**Response:** Disease/treatment knowledge was measured using eight questions taken from previous similar studies: ART reduces HIV related morbidity? ART reduces HIV related mortality? HIV is controlled by ART? does patient trusts the doctor? do you knows how to deal with side effects? do you stops taking ART on side effects without health professionals’ consultation, do you know the effectiveness of ART? and not abiding to ART leads to drug resistance? This ART knowledge score was calculated as a continuous variable by summing the participant’s number of correct responses to 8 statements. One point was awarded for each correct response (Yes or No for correct statement), and zero for each wrong response, with a maximum obtainable correct score of 8 for each respondent. The knowledge score was categorized into two levels indicated by poor knowledge/not knowledgeable (0-4) and good knowledge/knowledgeable (5-8). This is briefly discussed in this revised version of our manuscript.

20. The variable on social support is vague and undefined in methods. Social support from who? Family, friends, or personnel? Considering that true social support can only be evaluated in case of disclosure of status. Reported information should be very precise

**Response:** The social support measured in this study is a support which can be from families, friends, relatives or any other personnel. It was measured by Oslo 3-item social support scale as mentioned above. These items are:

How many people are so close to you that you can count on them if you have great personal problems?

1. None
2. 1-2
3. 3-5
4. >5

How much interest and concern do people show in what you do?

1. None
2. Little
3. Uncertain
4. Some
5. A lot

How easy is to get practical help from neighbors if you should need it?

1. Very difficult
2. Difficult
3. Possible
4. Easy
5. Very easy

Then, the patient is considered having “poor support” if the range is 3–8, “moderate support” if the range is 9–11 and “strong support” if the range is 12–14.

21. Another problem is what you called adverse drug reaction in your questionnaire. Please precise and clearly define all these variables in the methods section.

**Response:** The presence of adverse drug reaction was assessed by asking the patient whether he/she experienced any of them. If the patient developed at least one of the common side effects of ARV drugs, he/she was considered as having drug side effects. The question was asked as follows: “Have you experienced any of the side effects of ARV medication listed below?”

Loss of appetite	Rash or hypersensitivity

Lipodystrophy	Fatigue

Nausea and vomiting	Trouble sleeping

Diarrhea	Numbness in hands and legs

Mood changes	Sign and symptoms of anemia

If the patient experienced at least one of the side effects listed above, tick yes as the response to the question which indicate the patient has experienced adverse drug reactions. These are clearly described in the method section of this revised version of our manuscript.

22. The adherence status in this study is a very important proportion in this study. Please do present this proportion with its 95% confidence interval. In addition, it would be more light to present those with adherence evaluated to be between below 70%, between 70-80%, 80-90%, 0-95%... before those evaluated above 95%. This will help the reader understand the relative adherence level of the treated population.

**Response:** We didn’t indicate the adherence proportion with its 95% confidence interval in the first submission of our manuscript. However; we included in this revised version of our manuscript. Based on reviewer’s request, the detail proportion of adherence were explained in this revised version of our manuscript as follows: “The adherence rate was calculated by dividing the number of pills the patient actually taken by the number of tablets patients should have taken x100. About 3 (1.0%) of the study participants have adherence rate of < 70%, 7 (2.3%) of the study participants have adherence rate of 70-79.9%, 12 (3.9%) of the study participants have adherence rate of 80-89.99%, 60 (19.7%) of the study participants have adherence rate of 90-94.99% and 223 (73.1%) of the study participants have adherence rate of >/= 95%. Then, based on WHO guidelines, patients who reported intake of ≥ 95% of the prescribed medication were considered adherent; and those with a reported intake of < 95% were classified as non-adherent. Accordingly, out of the total of 305 study participants, 223 (73.1 %) were adherent to their medication (95% CI = 68.2, 78.0) and 82 (26.9 %) were not adherent to their medication (95% CI = 22.0, 31.8).”

Discussion
The English needs strengthening. This section is short and really does not put to context the findings in this research. The authors spend time comparing their findings on the ART adherence status to those in other studies and fail to discuss the true meaning and implication of their results. Some of the factors are addressed and others no, what criteria did the author use in choosing which factors to discuss and which not to. Discrepancies are not really well justified.

**Response:** We agree that the discussion part of our first submission was short. In this revised version of our manuscript, we discussed the meaning and implication of the finding in sufficient details. Some of the factors which initially didn’t addressed are discussed in this revised version. Possible justifications for discrepancies are also indicated.

Conclusion
Please reformulate the sentences and correct the English errors

**Response:** We reformulated our conclusion, and we hope the grammar errors are corrected in this revised version of our manuscript.

Tables
The results on the tables are well presented and can be followed but these statistical analyses are not well described in the data analysis section. Please correct accordingly. Also define abbreviations like COR, AOR, CI below your tables.

**Response:** The statistical analysis used in this study were not fully described in the previous version of our manuscript. We well described the statistical analysis used in this revised version of our manuscript. Abbreviations such as COR, AOR, CI were also defined.

Data availability statement is missing. The author should provide the data sets analyzed for the above findings.

**Response:** The data used during this study are available on request from the corresponding author. We sent you the data sets analyzed for the findings as independent file in the submission of this revised manuscript The data availability statement is also included in this revised version of our manuscript.

Thank you very much! I really appreciate your comments
