# Supplementary material for: Adherence to antiretroviral therapy and associated factors among Human immunodeficiency virus positive patients accessing treatment at Nekemte referral hospital, west Ethiopia, 2019
Source: PLoS One. 2020 May 11;15(5):e0232703. doi: 10.1371/journal.pone.0232703 (PMC7213685; doi:10.1371/journal.pone.0232703)
Supplement: Attachment — Submitted filename: Reviewer Comments.docx [file pone.0232703.s003.docx]

**Responses to questions**

1. The study presents the results of original research.

Yes

2. Results reported have not been published elsewhere.

I don’t know

3. Experiments, statistics, and other analyses are performed to a high technical standard and are described in sufficient detail.

Yes

4. Conclusions are presented in an appropriate fashion and are supported by the data.

Yes

5. The article is presented in an intelligible fashion and is written in standard English.

Yes,

6. The research meets all applicable standards for the ethics of experimentation and research integrity.

Yes

7. The article adheres to appropriate reporting guidelines and community standards for data availability.

Yes

Reviewer Comments

**Background**

1 Inclusion of a brief description of key elements of ART services offered at the hospital can make reader understand the context better.

### Sample size determination and sampling techniques

1. Brief description of the sampling method would help reader to understand and determine if there were on potential sources of bias that can affect results and interpretation. What was the sampling framework – ART records at the facility or patients were randomly selected as they came for their clinic visit during the study month?
2. 311 patients should be estimated sample size for the study
3. It is not clear if 311 patients represent total number of patients at the facility, in the study design and setting section, “*All ART patients on treatment follow up at* *Nekemte referral hospital was the source population and the sampled ART patients who had treatment follow up during the* *study period was the study population”,* It would be easy to follow the sampling methodology if total number of patients on ART is mentioned and how random sampling was applied.

**Ethical consideration**

“Filled out questionnaires were carefully handled and all access to results was kept strictly within the author” brief description how records were handled and stored can help the leader to understand instead of just “carefully handle and kept strictly by author”

**Results**

311 sampled - state out of how many patients in total.

How were social support categories derived?

What substances were used?

WHO clinical staging and CD 4 count levels- explain when these were determined for the patients as they are likely to be influenced by ART. It is not clear if this was the patient’s status at the time of the study or at the time of the ART initiation. Or how recent?

General comment: the author should ensure the grammatical errors are corrected throughout the manuscript.
